# Real-Time Feedback of the Applied Light-Curing Technique and Its Impact on Degree of Conversion of Composite Restorations—A Study with Undergraduate Dental Students

**DOI:** 10.3390/jpm11101012

**Published:** 2021-10-09

**Authors:** Phoebe Burrer, Matej Par, Thomas Attin, Tobias T. Tauböck

**Affiliations:** 1 Center for Dental Medicine, Department of Conservative and Preventive Dentistry, University of Zurich, Plattenstrasse 11, 8032 Zurich, Switzerland; thomas.attin@zzm.uzh.ch (T.A.); tobias.tauboeck@zzm.uzh.ch (T.T.T.); 2 Department of Endodontics and Restorative Dentistry, School of Dental Medicine, University of Zagreb, Gundulićeva 5, 10000 Zagreb, Croatia; mpar@sfzg.hr

**Keywords:** dental education, dental patient simulator, individual instructions, light polymerization, resin composite, degree of conversion

## Abstract

The objective of this study was to investigate the effect of individual instructions and training of dental students on the amount of applied light irradiance before and after training using a patient simulator with integrated visual feedback. Furthermore, the effect on the degree of conversion of composite restorations placed by the dental students was assessed. Forty-two dental students, split into two groups, light-cured a simulated restoration in tooth 27 of a dental patient simulator for 20 s. The irradiance (mW/cm^2^) received by the detector was measured in real-time before and after individual instructions and training, and the energy delivered (J/cm^2^) was calculated for each student. The degree of conversion at the bottom of incrementally placed composite restorations prior to individual instructions (group 1) and after individual instructions (group 2) was assessed using Fourier-transform infrared (FTIR) spectroscopy. The irradiance and degree of conversion measurements were re-assessed after all students received individual instructions. Data were analyzed using Wilcoxon signed-rank test and Mann-Whitney U-test at an overall level of significance of α = 0.05. A significant increase (*p* < 0.001) in applied light irradiance could be observed after individual instructions for both groups, with notably reduced data scattering. However, no significant difference was detected for the degree of conversion of placed composite restorations before or after instruction and training. Neither gender nor age of the dental students affected the obtained results. Consistent light energy delivered by dental students could be achieved through individual instructions and training with a patient simulator, also leading to less scattered irradiance results. However, the improved light-curing performance after the training did not affect the degree of conversion of the placed class II composite restorations.

## 1. Introduction

Photo-polymerizing a resin composite restoration is often promoted to be a simple and uncomplicated procedure, and dental light-curing devices have become an essential tool in dental practices. However, several studies have described uncertainties regarding the light-curing technique of resin composite, even for long-serving dentists [1,2]. It therefore seems indispensable to adequately instruct users of light-curing devices, to improve their performance and achieve reproducibly good results.

The successful placement of a resin composite restoration requires a fundamental knowledge of all processes and handling of the materials applied for the restoration [3]. Major aspects concern the light-curing device used by the practitioner, the light-curing time, and the thickness of the placed resin composite material [4,5,6,7,8,9]. A basic understanding and awareness of possible complications that may occur during the process of light-curing seems mandatory. One of the challenges in the light-curing procedure is the often-limited accessibility to the resin composite surface that is intended to be photo-polymerized. It is often challenging to position the light-curing tip as close and perpendicular to the composite surface as possible. Accordingly, studies recommend a prolonged light-curing time when surfaces are difficult to reach [1,9,10,11,12,13].

Dental patient simulators are available, which are equipped with a laboratory-grade spectro-radiometer to measure the delivered irradiance and energy in a simulated cavity. These simulators should help to evaluate and train dental students and interested professionals regarding optimization of their light-curing technique [14]. Individual instructions during light-curing application and immediate feedback are made possible by displaying the achieved light irradiance in real time with the patient simulator software.

To evaluate the quality of a composite restoration, degree of conversion (DC) or micro-hardness measurements are frequently used to estimate the effectiveness of light-curing at a certain layer thickness [15,16,17,18,19]. Moreover, adequate photo-polymerization and the physical properties of dental resin composites are mutually dependent [3,20]. Inadequate light-curing might result in a reduced degree of conversion, reduced bond strength, increases the risk for development of marginal gaps and may in general decrease the longevity of the placed composite restoration [3,15,16,21,22,23].

Improving the practitioner’s knowledge of light-curing must, therefore, be a designated aim of dental education [1,24,25], especially to support underperforming students [26]. Many of these important studies have investigated the influence of individual instructions and training on the light-curing performance of dental students. However, the question of whether such light-curing training would have an impact on the material parameter, expressed in terms of the degree of conversion of composite restorations, has not been addressed to date.

Therefore, the aim of the present study was to determine the light irradiance that dental students delivered to a simulated restoration during light-curing before and after individual instructions and training with the patient simulator, and to assess the degree of conversion of placed composite restorations also before and after the individual instructions. The tested hypotheses were that dental students would improve their light-curing technique after individual instructions and simulator-based training, resulting in (i) improved irradiance values and (ii) a higher degree of conversion of composite restorations placed by the students.

## 2. Materials and Methods

### 2.1. Test Groups

Forty-two students in their third year of dental medicine studies, attending the preclinical phantom course in Conservative Dentistry at the University of Zurich, were included in the study. Informed consent was obtained from all students. Participation was voluntary and had no influence on the evaluation of the students’ coursework. Study participants were anonymized using individual codes. A declaration of non-jurisdiction (BASEC-Nr. 2019-00649) was obtained from the Swiss Ethics Committees on research involving humans.

**Figure 1:**
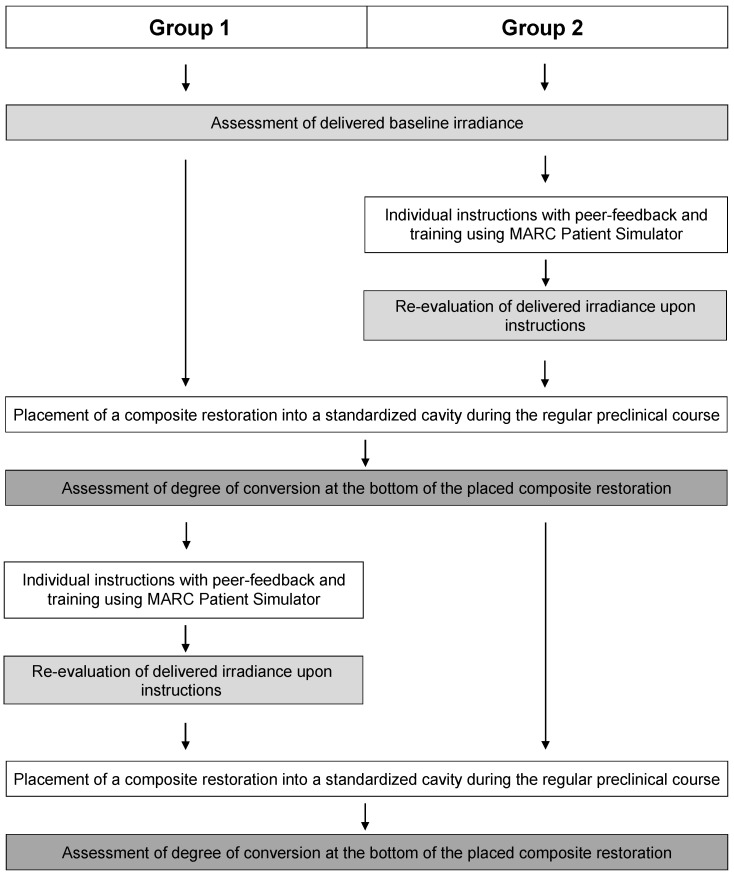
Experimental design and schedule.

The experimental design of the study and schedule for the practical parts are shown in Figure 1. At first, baseline irradiance measurements were collected for all dental students. In a second phase, the individual training and testing with the MARC Patient Simulator was performed for the respective group on the same day.

At the beginning of the preclinical phantom course, the dental students had a basic knowledge of light-curing from regular theoretical lectures, but little experience in its practical application. To assess their baseline light-curing performance, the irradiance delivered to a simulated class I restoration (tooth 27) in a dental patient simulator (Managing Accurate Resin Curing (MARC) Patient Simulator; BlueLight Analytics, Halifax, NS, Canada) was measured for each dental student. The MARC Patient Simulator consists of a manikin head equipped with an inside laboratory-grade spectrometer (USB4000; Ocean Optics, Dunedin, FL, USA), connected by a fiber-optic cable to a 3.9-mm diameter cosine-corrected light detector (CC3-UV; Ocean Optics, Dunedin, FL, USA). In this system, the light detector determining the emitted irradiance is positioned on the bottom of a left upper second molar (tooth 27), which contained a prepared class I cavity at a distance of 4 mm from the cusp tip and 2 mm below the cavosurface edge of the cavity. The irradiation time was set to 20 s and the same light-curing device (SmartLite Focus; Dentsply Sirona, Bensheim, Germany) was used throughout the whole study. The consistent irradiance output of the light-curing device (1150 mW/cm^2^) was monitored at regular intervals using a calibrated laser power meter paired with a compatible thermopile sensor (FieldMaxII-TO and thermopile PM2; Coherent, Santa Clara, CA, USA).

To simulate clinical conditions, the MARC Patient Simulator was attached to a dental chair and the mouth-opening was limited to 40 mm. The simulator was additionally equipped with a dental rubber dam, and a sectional matrice and separation ring (Palodent V3 Sectional Matrix System; Dentsply Sirona, Bensheim, Germany) were placed to better mimic and demonstrate the often reduced accessibility to a cavity. Protective orange eyeglasses, transparent protective glasses, an orange light shield, a mirror and a probe were freely selectable and usable in the light-curing process, and laid on a tray adhered to the dental chair. The dental students were asked to light-cure the simulated class I restoration for a period of 20 s. The irradiance (mW/cm^2^) delivered to the detector of the MARC Patient Simulator was recorded in real time, and the energy received per unit area (J/cm^2^) was automatically calculated by the software. 

### 2.2. Individual Instructions on Light-Curing with MARC Patient Simulator

After baseline measurements, the dental students were split into two groups. Group 1 consisted of seven male and fifteen female students aged between 21 and 34 years, and group 2 was composed of eleven male and nine female students aged between 21 and 27 years. The first group started directly with the hands-on part of placement of a composite restoration without receiving any instructions on light-curing (Figure 1). The second group of students (group 2) received immediate individual coaching and feedback, using the same light-curing device as before (SmartLite Focus; Dentsply Sirona, Bensheim, Germany). This theoretical and practical training section was divided into three parts [26]: First, the students were taught how to ideally adjust the dental chair and head of the patient simulator, considering ergonomic aspects. Students then received individual instructions on ideal photo-polymerization using the MARC Patient Simulator. They were shown how to correctly position the light-curing tip, in a perpendicular manner and as close as possible to the composite surface, with correct angulation of the light-curing tip to enable straight access to the restoration. Furthermore, they were advised to wear blue-light blocking orange protective eyeglasses during the curing process to allow for both the possibility of looking directly at the composite restoration and to have one hand free to stabilize the tip of the light-curing device to avoid trembling hand movements. Their attention was also drawn to the fact that incorrect positioning of the light-curing tip, or their hands holding and stabilizing the tip may lead to unfavorable shadow formation, reducing the amount of light that reaches the composite surface. The effects of these instructional advices were practically demonstrated with the MARC Patient Simulator, and real-time feedback was provided for the students to follow and to help them understand the consequences of small changes and flaws in the light-curing technique.

Following the first part of the practical demonstration, the second part of individual instructions and feedback was dedicated to the dental students for their training time. Here, students were asked to light-cure the simulated restoration of tooth 27 in the MARC Patient Simulator for 20 s to practice light-curing as shown before as often as they wanted. During this second part of the training, the irradiance profiles delivered by the dental students were analyzed, small mistakes could be corrected and suggestions on how to improve their light-curing performance could be tested immediately. When they felt confident, students were invited to exercise light-curing with the MARC Patient Simulator on their own, representing the third part of the individual training.

After the three-part training and feedback section, a re-evaluation of the instructions on the delivered irradiance was performed by the dental students under the same conditions as for the baseline measurements. The instructed dental students (group 2) were then asked to light-cure the simulated restoration of tooth 27 of the MARC Patient Simulator for 20 s, and the light-curing profile received by the detector was recorded in real time.

### 2.3. Placement of First Composite Restoration

After baseline irradiance measurements were taken for both groups, and re-evaluation upon the instructions was carried out by group 2, a hands-on part during the regular preclinical phantom course was performed, so that students could transfer and implement the techniques learned during their training on real composite restorations.

All dental students were asked to place a resin composite restoration in the dental manikin phantom head used for their regular class work. The dental manikin head was equipped with a dental training model (model series ANA-4; frasaco GmbH, Tettnang, Germany), with a prepared mesial class II cavity in tooth 27. This custom-designed cavity had a total height of 4 mm and was divided by a marking line at a height of 2 mm. The marking at 2-mm height was selected to make the incremental layering technique of composite restorations easier to visualize for the dental students. Each tooth was equipped with a specific number for internal recognition. In brief, the students’ task was to prepare the cavity for a composite restoration, including the placement of a dental rubber dam and a sectional matrix, acid etching with 35% phosphoric acid (Ultra Etch; Ultradent Products, South Jordan, UT, USA), and application of an adhesive system (OptiBond FL; Kerr, Orange, CA, USA). Each application step was audited by an instructor of the preclinical class on an attestation paper. Placement of the resin composite restoration was requested to be performed by two horizontal composite increments. The first increment of the nano-hybrid composite material (Ceram.x universal; Dentsply Sirona, Bensheim, Germany) had to be placed up to the height of the marking groove at 2 mm and photo-polymerized for 20 s with the same light-curing device as used with the MARC Patient Simulator. The second increment also had a thickness of 2 mm, according to the guidelines for the maximum incremental layer thickness of the composite used, and was subsequently light-cured for 20 s. The students were then asked not to polish the composite restoration in order to prevent excessive water absorption and possible heating of the composite material through polishing measures. Afterwards, the composite restorations were handed to the instructors. The composition of the composite material is shown in Table 1.

### 2.4. Individual Instructions with MARC Patient Simulator of Group 1 and Placement of Second Composite Restoration for Both Groups

After all dental students in both groups had completed the placement of composite restoration, the first half of the dental students (group 1), which had not been instructed at this point, received the same individual instructions and three-part training with the MARC Patient Simulator as group 2 had received before (Figure 1). Irradiance measurements were then performed, and the delivered energy was recorded in real time for 20 s of light-curing. At this point, all dental students in both groups had been instructed on the light-curing technique by means of the MARC Patient Simulator. 

Then, the second hands-on section was performed, again during regular preclinical class. The procedure remained the same as in the first run. As the placement of composite restorations was performed during regular class, and to maintain equality for all students, both groups of dental students were asked to place a second resin composite restoration in another prepared class II cavity of tooth 27 (Figure 1). Consequently, for group 1, the placement of composite restorations was performed before and after the instructions on light-curing. However, for students in group 2, this was the second placement of a composite restoration after instructions and training with the MARC Patient Simulator, prior to the degree of conversion assessment of all placed composite restorations.

### 2.5. Degree of Conversion Analysis

Degree of conversion (DC) was evaluated using Fourier-transform infrared (FTIR) spectroscopy (Cary 630 FTIR; Agilent Technologies, Santa Clara, CA, USA) with an attenuated total reflectance (ATR) device containing a ZnSe crystal. Per specimen, 64 scans were taken at a resolution of 4 cm^−1^ and the recorded spectra ranged from 650 to 4000 cm^−1^. Spectra of uncured composite material were taken, and a background spectrum was recorded prior to each measurement. Degree of conversion calculation was performed from the changes in the ratio of absorbance intensities (Abs) of the aliphatic C=C spectral bands at a peak height of 1636 cm^−1^, and the aromatic C–C bands at a peak height of 1608 cm^−1^ (internal standard) between the polymerized and unpolymerized specimens according to the following Equation (1) [15,27]:

(1) $$\mathrm{DC}\;\left(\%\right)=[1-\frac{{{[\mathrm{Abs}\;(1636\;\mathrm{cm}}^{-1}{)\;/\;\mathrm{Abs}\;(1608\;\mathrm{cm}}^{-1})]}_{\mathrm{cured}}}{{{[\mathrm{Abs}\;(1636\;\mathrm{cm}}^{-1}{)\;/\;\mathrm{Abs}\;(1608\;\mathrm{cm}}^{-1})]}_{\mathrm{uncured}}}]\;\times \;100,$$

For FTIR measurements, the teeth containing the composite restoration were first adhered with the opposite surface on a sample holder (Wenka, Karl Wenger SA, Courgenay, Switzerland) and polished with a 4000-grit silicon carbide grinding paper (Buehler-Met II; Buehler, Esslingen, Germany) in a polishing machine (Planopol-2; Struers, Ballerup, Denmark) under constant water-cooling. Then, specimens were cut parallel to the sample holder and placed on the ZnSe crystal. Three FTIR measurements were performed per specimen in one line at 4-mm depth of each composite restoration, and the mean value was calculated.

### 2.6. Statistical Analysis

Shapiro-Wilk’s test for normality of data distribution and Levene’s test for homogeneity of variances were performed. As data were not normally distributed, non-parametric testing was employed. Intra-group and inter-group comparisons were performed for irradiance measurements as well as for the degree of conversion of the placed composite restorations. For intra-group comparisons between the two timepoints, data were analyzed using Wilcoxon’s signed-rank test. For inter-group comparisons within a given timepoint, Mann-Whitney U-test was used. Additionally, Mann-Whitney U-tests for independent samples were performed to assess the impact of gender on the outcome variables. The impact of age was assessed using Spearman’s correlation. All statistical analyses and plots were performed using SPSS version 27 (IBM Corp. Armonk, NY, USA), and the level of significance was set to α = 0.05. 

## 3. Results

### 3.1. Light Irradiances before and after Individual Instructions

**Figure 2:**
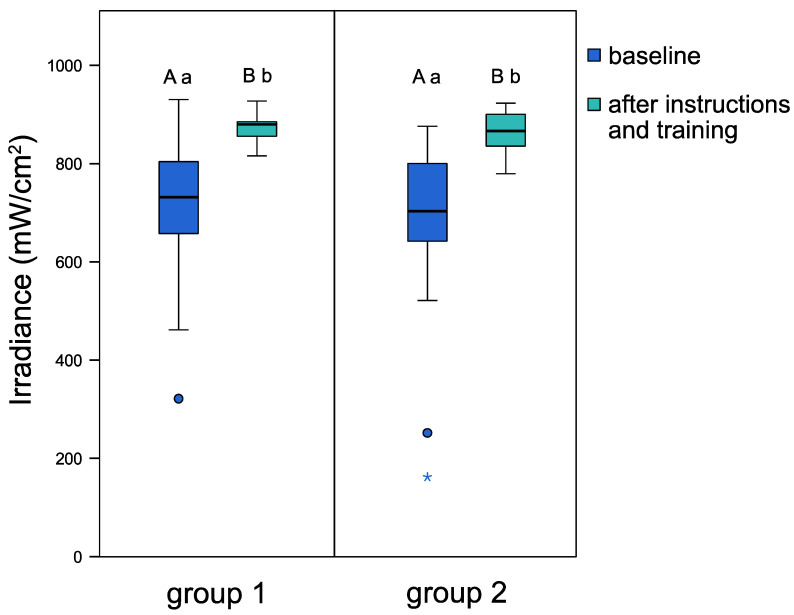
Light irradiance (mW/cm^2^) achieved by the two groups of dental students at baseline and after individual instructions and training with the MARC Patient Simulator. The boxplots show the medians (bold black lines) and the boxes represent the 25% and 75% data quartiles, whereas the whiskers represent 1.5 × interquartile range (IQR), or minima and maxima of the distribution if below 1.5 × IQR; outliers are shown by circles, and extreme outliers are presented by asterisks. Groups marked with different uppercase letters (intra-group comparisons) or different lowercase letters (inter-group comparisons) are significantly different from each other (*p* < 0.05).

Figure 2 illustrates the light irradiances before and after individual instructions on light-curing of both groups. At baseline (before instructions), both groups delivered similar light irradiances. After individual instructions and training with the patient simulator, both groups attained a significant increase in delivered light transmittance. Median values increased from 731.7 mW/cm^2^ to 879.8 mW/cm^2^ for group 1 (*p* < 0.001), and from 703.2 mW/cm^2^ to 866.3 mW/cm^2^ for group 2 (*p* < 0.001). Considerably lower data scattering was observed after instruction for both group 1 and group 2, with a 4.7-fold and 2.7-fold lower interquartile range (IQR), respectively, when compared to baseline IQR. The demographic components of gender and age had no significant influence on the obtained light irradiance values.

### 3.2. Degree of Conversion of the Composite Restorations

**Figure 3:**
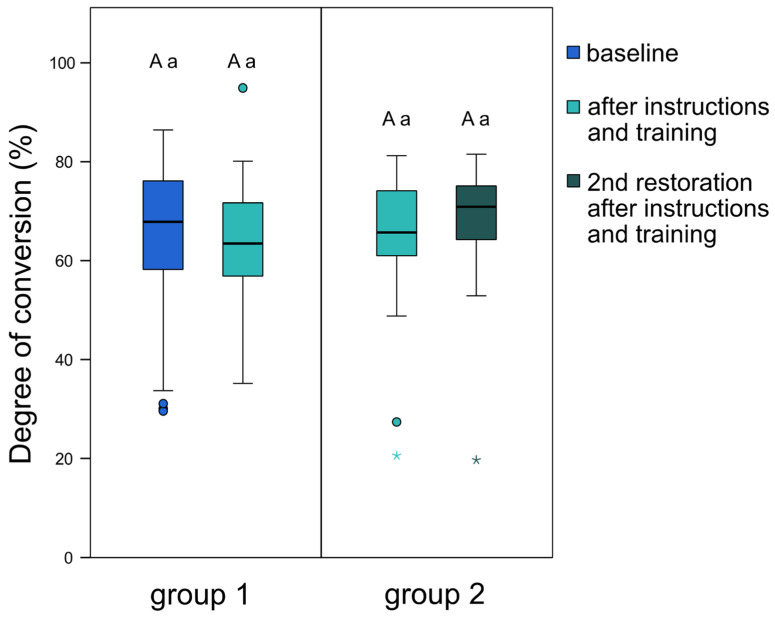
Degree of conversion (%) of the composite restorations of group 1 and group 2, measured at the bottom of the 4-mm-thick composite restorations. For group 1, degree of conversion was assessed of composite restorations placed before and after individual instructions and training; for group 2, both degree of conversion assessments were performed on composite restorations placed after individual instructions and training. The boxplots show the medians (bold black lines), and the boxes represent the 25% and 75% data quartiles, whereas the whiskers represent 1.5 × interquartile range (IQR), or minima and maxima of the distribution if below 1.5 × IQR; outliers are shown by circles, and extreme outliers are presented by asterisks. Groups marked with different uppercase letters (intra-group comparisons) or different lowercase letters (inter-group comparisons) are significantly different from each other (*p* < 0.05).

Figure 3 depicts the degree of conversion of the composite restorations placed by the dental students. For group 1, no significant difference could be detected in degree of conversion before and after individual instructions and training. Furthermore, no significant difference could be noted between group 1 and group 2 after both groups had completed their training session with the patient simulator. For group 2, a slight, yet not statistically significant, increase in median degree of conversion was observed from the first to the second placed composite restoration. Neither gender nor age significantly affected the degree of conversion of composite restorations placed by dental students.

## 4. Discussion

The results of the present study revealed a significant improvement in energy delivered by dental students after patient simulator-based training and feedback, although this did not affect the degree of conversion of placed composite restorations. Despite the high success rates for posterior teeth [4,6,28,29], failure in composite restorations might occur, and proximal gingival margins of class II restorations are at especially high risk of the development of secondary caries [30,31]. The material used and its application, as well as the process of light-curing, remains highly operator-dependent and have been shown to have a major impact on the longevity and quality of composite restorations [4,5,6,32,33,34].

After attending individual instructions and training with the MARC Patient Simulator, dental students in both groups attained significantly higher light irradiance values than at baseline without instructions (*p* < 0.001). Thus, the first tested hypothesis was accepted. At baseline measurements, the energy delivered by dental students ranged widely, between a minimum of 3.2 and a maximum of 18.5 J/cm^2^. However, after peer-feedback training, more homogenous values, ranging between 16.5 and 18.5 J/cm^2^, were achieved. Similar results were obtained by previous studies, indicating the beneficial effect of light-curing instructions to achieve and maintain a sufficient and consistent light-curing performance [1,14,26,35,36,37]. In the present study, it was observed during baseline measurements that dental students delivering lower light irradiance values did not pay proper attention to the correct positioning of the light-curing tip and looked away during the procedure. After training from the instructor about the advantages of wearing orange protective glasses, irradiance values improved significantly, resulting in less scattered data after individual training with the MARC Patient Simulator.

However, the improved light irradiance values did not affect the degree of conversion of the composite restorations placed by the dental students. Thus, the second hypothesis was rejected. Surprisingly, no significant difference was found in degree of conversion between the first (before instructions on light-curing) and second (after instructions on light-curing) placed composite restoration of group 1, or between the two groups. However, it was observed that the composite restorations of some students with an initially lower light-curing performance also achieved a considerably lower degree of conversion. However, it can only be assumed that the same student light-curing the composite restoration might have delivered the same total energy as during the evaluation at the MARC Patient Simulator. The obtained values increased after the individual light-curing instructions, again emphasizing the importance of individual instructions on correct photo-polymerization, especially for underperforming students. Moreover, a slight increase in degree of conversion was observed for students in group 2, with two consecutive placed composite restorations after instructions and training, which indicates a further beneficial effect of light-curing training on repeatedly placed composite restorations. Those dental students were able to retain their improved light-curing technique and achieved similar or an even higher degree of conversion for the second placed composite restoration.

Additionally, the energy delivered by most of the dental students seems to be more than sufficient to adequately cure the composite material, reaching up to 18 J/cm^2^. As an emitted energy of 10 J/cm^2^ is generally considered sufficient to reach an acceptable degree of conversion at the restoration bottom [14,38], this specified minimum value was mostly already attained or exceeded at baseline measurements. This “over-exposure” of delivered energy may be the main reason for the similar degree of conversion values and probably compensated for eventual flaws during light-curing, resulting in the restorations reaching the maximum attainable degree of conversion for the composite material used, regardless of students’ inconsistencies in light-curing. The differences in the degree of conversion due to variations in curing technique might have been more pronounced if a shorter light-curing time was applied. 

Despite the greatest care being taken when conducting the present study, a possible limitation is that students may have paid more attention to their light-curing technique because they knew they were participating in a study. This could have affected both irradiance results, as well as the degree of conversion of the placed composite restorations. Still, patient simulator-based training and individual instructions significantly improved the light-curing technique of dental students and established a more reliable light-curing performance, which should be beneficial in their daily clinical practice, and ensure consistently good light-curing performance and the longevity of composite restorations.

## Figures and Tables

**Table 1:** Composition and manufacturer details of the composite material used in the present study.

Product	Composition	Lot No.	Manufacturer
Ceram.x universal	Matrix: methacrylic polysiloxane nanoparticles, poly-urethane-methacrylate, Bis-EMA ^1^, TEGDMA ^2^, dimethacrylate resin, ethyl-4-(dimethylamino)-benzoate Filler: spherical, pre-polymerized SphereTEC fillers (d3,50 ≈ 15 μm), non-agglomerated barium glass, ytterbium fluoride, camphorchinone Filler content: 77–79 wt%, 59–61 vol%	1801000916	Dentsply Sirona, Bensheim, Germany

^1^ Bis-EMA: ethoxylated bisphenol-A-dimethacrylate, ^2^ TEGDMA: triethylene-glycol-dimethacrylate.

## Data Availability

The datasets generated and analyzed during the current study are available from the corresponding author on reasonable request.
